# Anatomical factors affecting the time required for microsurgical subinguinal varicocelectomy

**DOI:** 10.1186/s40064-016-2689-0

**Published:** 2016-07-08

**Authors:** Jung Keun Lee, Ho Young Ryu, Jae-Seung Paick, Soo Woong Kim

**Affiliations:** Department of Urology, College of Medicine, Seoul National University, 110 Daehak-ro, Jungno-Gu, Seoul, 110-744 Republic of Korea

**Keywords:** Microanatomy, Operation time, Subinguinal varicocelectomy, Surgical difficulty, Varicocele

## Abstract

**Background:**

Microsurgical subinguinal varicocelectomy (MSV) is considered an effective and less morbid procedure, but the difficulty in preserving testicular arteries is a limitation of this procedure. We identified the microanatomy encountered during MSV and clarify its significance to the difficulty of the procedure.

**Methods:**

Three hundred and twenty-six patients who underwent left MSV were evaluated. Detailed intraoperative microanatomy was recorded for each case. A classification system was used to assess the anatomical relationship between the internal spermatic artery and the varicose veins as follows: type I (non-adherent to the veins), type II (adherent to the veins), and type III (surrounded by veins). Type III cases were further divided into types III-a (an arterial pulse) and III-b (a blurred arterial pulse). A linear regression analysis of the factors associated with the length of the operation was used to determine the difficulty of the surgery.

**Results:**

A mean number of 8.2 internal spermatic veins were ligated. Internal spermatic arteries were classified as type I in 14 % of patients, type II in 57 %, and type III in 29 % (III-a in 20 % and III-b in 9 %). A large number of internal spermatic veins and higher internal spermatic artery type were observed significantly more often in grade 3 varicoceles (p < 0.05). The types of internal spermatic arteries (ρ = 0.458) and numbers of internal spermatic veins (ρ = 0.431), cremasteric veins (ρ = 0.197), and gubernacular veins (ρ = 0.119) were significantly associated with the length of the operation (p < 0.05).

**Conclusions:**

Anatomical factors were associated with the varicocele grade and surgical difficulty. These findings are helpful to perform MSV.

## Background

A varicocele is defined as an abnormally dilated spermatic and scrotal vein, and is a left-dominant disorder (Gargollo and Diamond 2009). Treatment of the varicocele is generally considered in patients with infertility, and other indications for treatment are testicular pain or testicular size discrepancy (Choi and Kim 2013; Diamond et al. 2011). Although varicoceles are repaired by a variety of surgical approaches, the most important principle of varicocelectomy is to ligate all veins except one, and to preserve all arteries and lymphatics. According to this principle, microsurgical varicocelectomy is recommended as the gold standard for varicocele repair (Mirilas and Mentessioud 2012). The advantages of microscopic subinguinal varicocelectomy (MSV) are that it is less painful and allows patients to recover more easily than the inguinal approach (Mirilas and Mentessioud 2012; Hopps et al. 2003). However, MSV has a weakness in that it is more difficult to perform than an operation via an inguinal approach, thus it requiring additional surgical training (Hopps et al. 2003).

The surgeon must ligate more veins in the subinguinal approach than in the inguinal approach, and more than one internal spermatic artery (ISA) is usually observed during MSV (Hopps et al. 2003). Isolation of the ISA can also be difficult owing to arterial compression by the external oblique aponeurosis and it is surrounded by a dense network of veins (Hopps et al. 2003). We believe that identifying and analyzing potential factors affecting the surgical difficulty is important to overcoming the surgical difficulty of MSV. However, surgeon-related factors vary and are improved by experience. Beyond surgeon-related factors, the anatomical factors of the varicocele affect the difficulty of MSV. In particular, difficulty separating the ISA from veins is a major issue during MSV. Thus, a more detailed description of how to separate the ISA from the adjacent veins can help to decrease the difficulty of MSV.

The objectives of the current study were to propose a classification system for separating the ISA from the veins based on its anatomical relationship to varicose veins and to evaluate the relationships among the anatomical factors and the length of the operation estimating the difficulty of the surgery.

## Results

Mean age of the patients at varicocelectomy was 28.0 ± 7.6 years. MSV was performed on patients the following indications: 116 patients (35.6 %) underwent varicocelectomy for infertility only, 125 (38.3 %) for testicular pain, 71 (21.8 %) for infertility and testicular pain, and 14 (4.3 %) because they were referred with an asymptomatic testicular mass. Varicoceles were graded as follows: grade I in 1.5 %, grade II in 17.5 %, and grade III in 81.0 %. Postoperatively 305 patients (93.6 %) returned for followed-up with a median 4 months (interquartile range 1 to 8). Ten patients had a scrotal swelling which reduced spontaneously. None required further surgical intervention. No cases of persistent or recurrent varicocele were found, and there was no testicular atrophy or persistent hydrocele formation on scrotal examination.

On average, we ligated 0.8 perforating external spermatic, 2.1 gubernacular, and 2.4 cremasteric veins per cord. A mean of 8.2 ISVs were ligated per cord. Single and multiple ISAs were identified and preserved in 61.3 and 38.7 % of cases, respectively. The majority of MSV cases had an ISA type II, and 19.6 and 9.5 % were type III-a and III-b, respectively. The patients were classified into two groups according to their varicocele grade (grade 1–2 vs. grade 3). Significant differences were found between these groups in terms of the number of ISVs, the ISA type, and the presence of an additional vasal vein (see Table 1). Intraoperative repairs were performed successfully in 4 patients with ISA injuries (2 in ISA type II and 2 in type III-a).

**Table 1:** Microanatomy encountered during microsurgical subinguinal varicocelectomy

Variable	Entire cohort	Grade 1–2	Grade 3	p value
Number	326	62	264	
Perforating ESV (%)	186 (57.1)	32 (51.6)	154 (58.3)	0.112
Gubernacular vein (%)	283 (86.8)	54 (87.1)	229 (86.7)	0.941
Number of gubernacular veins	2.1 ± 1.2	1.9 ± 1.1	2.1 ± 1.3	0.115
Number of ISVs	8.2 ± 2.3	7.5 ± 1.7	8.3 ± 2.4	0.022
Number of ISA (%)				
1 artery	200 (61.3)	37 (59.7)	163 (61.7)	0.871
2 arteries	113 (34.7)	23 (37.1)	90 (34.1)	
3 arteries	13 (4.0)	2 (3.2)	11 (4.2)	
ISA type (%)				
I	45 (13.8)	16 (25.8)	29 (11.0)	0.018
II	186 (57.1)	33 (53.2)	153 (58.0)	
III-a	64 (19.6)	8 (12.9)	56 (21.2)	
III-b	31 (9.5)	5 (8.1)	26 (9.8)	
Number of lymphatics	5.0 ± 1.2	4.8 ± 1.3	5.1 ± 1.2	0.120
Additional vasal vein (%)	82 (25.2)	9 (14.5)	73 (27.7)	0.032
Number of cremasteric veins	2.4 ± 0.9	2.2 ± 0.7	2.5 ± 1.0	0.094
Number of cremasteric arteries	1.5 ± 0.7	1.5 ± 0.9	1.6 ± 0.8	0.526

*ESV* external spermatic vein, *ISA* internal spermatic artery, *ISV* internal spermatic vein

The mean length of the operation was 82.4 ± 17.8 min (range 70.0–90.0 min). Four variables were shown to be significantly associated with the length of the operation (see Table 2). The most significant correlation was between the length of the operation and the ISA type (with the length of the operation increasing from type I to type III-b) (ρ = 0.458, p < 0.001). A multivariate linear regression model of the length of the operation using the four variables found to be significant in the Spearman rank analyses showed that the length of the operation increased with increasing ISA types and increasing the number of ISVs, cremasteric veins, and gubernacular veins (all p < 0.05). The coefficient of determination of the multivariate model was r^2^ = 0.293.

**Table 2:** The results of Spearman rank correlation analyses with the length of the operation used as the dependent variable

Variable	Correlation coefficient	p value
Number of perforating ESVs	−0.015	0.786
Number of gubernacular veins	0.119	0.032
Number of ISVs	0.431	0.001
Number of ISAs	0.059	0.286
Number of cremasteric arteries	0.082	0.140
Number of cremasteric veins	0.197	0.001
Number of lymphatics	−0.028	0.611
ISA types	0.458	0.001

*ESV* external spermatic vein, *ISA* internal spermatic artery, *ISV* internal spermatic vein

## Discussion

Information about the intraoperative anatomy is of fundamental importance during MSV which requires meticulous dissection. In the present study, we showed that the anatomy of the subinguinal approach is complex, and some anatomical characteristics were significantly associated with surgical difficulty. We proposed classifying the ISA types. Our data showed that the ISA type, the number of ISVs, the number of cremasteric veins, and the number of gubernacular veins were significantly correlated with the length of the operation. The ISA type showed an especially high correlation, which indicated time-limiting procedures during MSV. These results provide an insight on the complexity of microanatomy which adds to the technical difficulties of MSV in order to intraoperative injuries. Microanatomies vary substantially within patients. Considering this anatomic basis during MSV should result in good intraoperative as well as in good postoperative outcomes.

Although ligation of the ISA in varicocelectomy is a controversial issue (Salem and Mostafa 2009), Raman et al. observed that the caliber of the ISA was larger than the sum of the cremasteric and vasal arteries (Raman and Goldstein 2004). Furthermore, Chan et al. (2005) reported that ligation of the ISA is associated with an increase in testicular atrophy and a decrease in postoperative paternity.

Although preservation of the ISA is a key step in MSV, however it is not an easy task due to the multiple ISAs and the complex anatomical relationship between the ISAs and adjacent ISVs (Hopps et al. 2003; Libman et al. 2010; Lv et al. 2015). We found multiple ISAs in 39 % of our MSV procedures. Hopps et al. (2003) reported that 43 % of their MSV cases had multiple ISAs and Libman et al. (2010) reported that 50 % of their MSV cases had multiple ISAs. Similarly, Lv et al. (2015) reported that multiple ISAs were identified in 58 % of patients in their varicocele unit. Most studies have only reported the mean number of ISAs, thus it was unclear whether the number of ISAs is associated with the difficulty of the surgery. Our present results showed that the number of ISAs was not significantly associated with the difficulty of surgery.

Instead, we found that the ISA type was significantly associated with the difficulty of the procedure. Hopps et al. (2003) reported that 5 % of ISAs were located outside of the surrounding ISVs, whereas 95 % of ISAs were inside veins, and Lv et al. (2015) reported that 12 % of ISAs were isolated and 88 % were adherent. In our study, the ISAs were described in greater detail. We observed that 14 % of ISAs were isolated (type I), 57 % were adherent (type II) and 29 % were surrounded by the varicose veins (type III). When a type III ISA is present, more aggressive manipulation of the internal spermatic vessels was required. In particular, ISA type III-b requires more meticulous dissection than ISA type III-a, it is difficult to ligate all of the veins and splice those surrounding the ISA. Notably, we found that higher classifications of the ISAs were detected more frequently in the higher grade (especially grade 3) varicoceles.

Hopps et al. (2003) reported that the number of ISVs was significantly associated with varicocele grade. However, Libman et al. (2010) reported that there were no significant associations between the varicocele grade and microanatomy. By contrast, Belani et al. (2004) found that a higher varicocele grade was associated with a larger number of ISVs. We found that the number of ISVs was larger in grade 3 than in the lower grades. The number of ISVs was also associated with the length of the operation in the present study. Our findings suggest that more ISVs will be found and the surgical difficulty will be increased in grade 3 of varicoceles.

Another significant finding was the association between the increased operation time and the number of gubernacular and cremasteric veins. In the current study, examination of the gubernaculum was performed through the subinguinal approach without any exceptions. Despite the potential risk of scrotal hematoma and an increase in the length of the operation, a thorough examination of the gubernaculum is associated with lower rates of varicocele recurrence (Ramasamy and Schlegel 2006; Schiff et al. 2005). We also believe that ligation of the gubernacular vein is essential to prevent varicocele recurrence.

In our study, a mean number of 2.4 cremasteric veins were ligated and a mean number of 1.5 cremasteric arteries were identified. Although the cremasteric artery is not essential for supplying blood to the testis, we attempted to preserve all fine cremasteric arteries during MSV. The number of cremasteric veins had a significant correlation with the length of the operation. The reason for this correlation might be that most cremasteric arteries stick closely to large cremasteric veins, thus it requiring more time to preserve and separate the artery from the vein.

The potential limitations include the retrospective study design, although the description of the microanatomy was completed immediately after the operation for every case. Additionally, our data were based on the experience of a single surgeon who had performed various microsurgeries, thus these findings may not be applicable to inexperienced surgeons. However, the use of anatomical descriptions of all MSVs provided by a single surgeon at a single center can prevent the risk of bias with regard to the inter-surgeon variability and the learning curve. We did not evaluate the right varicocele and recurrent varicoceles, which have non-dominant patterns in the clinical setting, to avoid any potential bias.

## Conclusions

The microanatomy encountered during MSV was associated with the difficulty of the procedure. Especially, the type of ISA and the number of ISVs were associated with varicocele grade and the length of the operation for MSV. If a patient presents with a grade 3, the micro-surgeon should expect to the difficulty of surgery.

## Methods

### Study population

After institutional review board approval, we performed this study using patients who underwent left MSV by a single surgeon at our hospital between August 2005 and May 2015. All varicoceles were diagnosed based on a physical examination, and 326 cases were enrolled in this study after exclusion. Exclusion criteria were patients younger than 19 years, those with a history of left inguinal surgery, and those who underwent redo varicocelectomy. Varicoceles were graded according to Dubin and Amelar’s system as grade 1 (palpable only during the Valsalva maneuver), grade 2 (palpable at rest, but not visible), and grade 3 (visible and palpable at rest)(Dubin and Almelar 1970). All scrotal examinations were performed by a single physician. We did not routinely perform Doppler ultrasound pre- or post-operatively. All patients were followed-up 3 months after operation.

### Surgical methods and description of the microanatomy

With the patient under general anesthesia, we made a 2- to 3-cm subinguinal skin incision and the spermatic cord was identified. The spermatic cord was grasped by a Babcock clamp and was placed over a Nelaton catheter (no. 6). Then the testicle was delivered and the gubernaculum was examined in all cases. The surgeon found and ligated all perforating external spermatic and gubernacular veins larger than 2 mm in diameter. Any veins less than 2 mm in diameter were controlled by electronic cautery. Testis was returned to the scrotum. The external and internal spermatic fascia was incised, and the cord layers were separated by blunt dissection. A Nelaton catheter (no. 5) was then introduced between the internal spermatic contents (i.e., the vessels and lymphatics) and the external spermatic fascia and its associated structures, including the vas deferens. The internal spermatic vessels were carefully spread using the surgeon’s thumb with wet-gauze. We dissected the internal spermatic contents using an operating microscope (Carl Zeiss, Inc. Thornwood, NY, USA). Subtle pulsations usually showed the location of the ISA. We did not use a solution such as papaverine or intraoperative Doppler to identify the ISA. Once the ISA was dissected free of adherent veins, it was encircled with a vessel loop for identification. If subtle pulsation of the ISA was not noted, the internal spermatic veins (ISVs) larger than 2 mm were carefully dissected and ligated with 4–0 silk. During the dissections, the spermatic cord was skeletonized, and all ISAs and lymphatics were preserved and counted. We then elevated the external spermatic fascia and its associated structures. The vas deferens was dissected, and one vasal artery and one vasal vein were preserved. Additional vasal veins larger than 2 mm were also ligated. Subsequently, we dissected the remnant external spermatic contents. Any cremasteric arteries were preserved, and cremasteric veins larger than 2 mm were ligated.

**Fig. 1:**
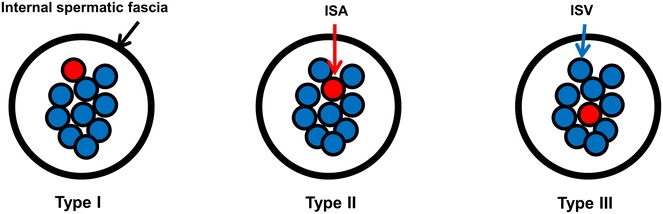
Cross-section of the internal spermatic cord showing various ISA types

After all procedures were completed, the number of ligated veins, preserved lymphatics, and preserved arteries were recorded. The anatomical relationship between the ISA and adjacent veins was classified as follows (see Fig. 1): type I (the ISA was located superficially and was non-adherent to the veins), type II (the ISA adhered to the veins) and type III (the ISA was surrounded by varicose veins). ISA type III was sub-classified further by the arterial pulsatility: III-a (an arterial pulse) and III-b (a blurred arterial pulse). When two or more independent ISAs were observed, a higher ISA type was selected among multiple arteries. The length of the operation was measured from the skin incision to skin closure.

### Statistical methods

Categorical variables were expressed as numbers with the percentages and numerical values were expressed as a group mean with standard deviations. Chi-squared and Fisher’s exact test were used to compare the microanatomy in the subgroups. The length of the operation was used to estimate the difficulty of the surgery. Spearman rank correlation analyses were used to determine the relationships between the microanatomy variables and the length of the operation. All statistical analyses were conducted using IBM SPSS Statistics 21.0 (SPSS, Inc., an IBM Company, Chicago, USA). p < 0.05 were considered statistically significant.

## Abbreviations

ESV: external spermatic vein
ISA: internal spermatic artery
ISV: internal spermatic vein
MSV: microsurgical subinguinal varicocelectomy
